# Characterization of Lipizzan Horse Population From Lipica Stud Using Molecular and Pedigree Data

**DOI:** 10.1002/age.70182

**Published:** 2026-08-12

**Authors:** Tamara Ferme, Minja Zorc, Marko Cotman, Matjaž Mesarič, Peter Dovč

**Affiliations:** ^1^ University of Ljubljana Biotechnical Faculty Ljubljana Slovenia; ^2^ University of Ljubljana Veterinary Faculty Ljubljana Slovenia

**Keywords:** genomics, Lipizzan horse, microsatellites, pedigree, selection signature, SNP array

## Abstract

In this study, we analysed genetic diversity, population structure and inbreeding in the Lipica Stud Farm population by integrating pedigree records, microsatellite (STR) and genome‐wide SNP data generated using the GGP Equine 70K BeadChip. The dataset comprised 233 horses from the Lipica Stud Farm, which served as the reference population for comparisons of diversity estimates based on pedigree‐, STR‐ and SNP data. The STR data were originally generated for routine parentage verification and were included here to compare inbreeding estimates based on pedigree, STR and genome‐wide SNP data. Pedigrees of the analysed horses were traced back to the 18th century, with the longest ancestral path spanning 35 generations. The mean pedigree inbreeding coefficient in the reference population was 11.55%, while ancestral inbreeding coefficients ranged from 5.93% to 15.42%. Microsatellite analyses revealed an average observed heterozygosity (Ho) of 0.68 and a mean of 5.9 alleles per locus, with STR‐based inbreeding (*F*_STR_) ranging from 0.10 to 0.15. However, genomic inbreeding estimated from runs of homozygosity (*F*_ROH_) varied between 0.09 and 0.28, with an average of 0.16. Selection signature analysis identified several putative candidate regions, whereas integrated haplotype score (iHS) analysis highlighted three candidate protein‐coding genes (*ZNF114*, *DENND5A,* and *TENM4*). The ROH islands contained 24 genes previously associated with pigmentation, reproduction, muscle function, and other biologically relevant traits in horses. These results provide a comprehensive genomic characterisation of the Lipica Stud Farm population and offer important insights into genetic diversity and inbreeding patterns in the Lipizzan breed.

## 1 Introduction

The Lipizzans are one of the oldest horse breeds in Europe and represent an important part of European cultural and equestrian heritage. Its history dates back to 1580, when Archduke Charles II established the stud farm in Lipica, located in present‐day Slovenia (Nürnberg 1993). The founder population consisted of nine stallions and 24 mares imported mainly from Spain, together with several local Karst mares (Dovc et al. 2006). Later introductions of Neapolitan, Andalusian, Barb, Frederiksborger, Kladruber and Arabian horses further contributed to the genetic background of this baroque horse breed (Clutton‐Brock 1999; Achmann et al. 2004).

For more than two centuries, breeding of Lipizzan horses was concentrated in a small number of court studs within the Habsburg monarchy, including Lipica, Kladrub and Kopčany, which formed a connected breeding system that shaped the genetic structure of the breed (Zechner et al. 2001). During the establishment phase of the breed, the first stallion lines and mare families were formed (Grilz‐Seger and Druml 2011). Today, the Lipizzan breed is based on six classical stallion lines (Pluto, Conversano, Neapolitano, Maestoso, Favory and Siglavy) and 17 maternal family lines that are maintained across several European studs (Achmann et al. 2011). In addition, an 18th mare family, Rebecca/Thais, was established in Lipica after 1947 (Rus 2011). Despite historical disruptions caused by wars and political changes, including the evacuation of the Lipica herd during the Napoleonic wars and both World Wars, the population was repeatedly re‐established through exchange of breeding animals among Lipizzan studs (Dovc et al. 2006). These exchanges were part of the traditional breeding system of the breed, in which classical stallion lines and mare families were maintained.

Today, 11 state stud farms in nine European countries manage breeding programmes aimed at preserving this unique genetic resource (Zechner et al. 2002; Achmann et al. 2011; Grilz‐Seger, Druml, et al. 2019). Because the Lipica stud represents the historical nucleus population of the breed, it provides an important reference for studying the genetic diversity and population structure of Lipizzan horses.

Several molecular approaches have been used to investigate the genetic history of the Lipizzan breed. Mitochondrial DNA (mtDNA) studies have been particularly useful for analysing maternal lineages. Kavar et al. (1999) investigated mtDNA sequence variation in the 16 classical Lipizzan mare family lines and identified haplotypes belonging to three main haplogroups (C1, C2 and C3). The diversity of mitochondrial haplotypes reflects the contribution of different ancestral breeds and indicates that the Lipizzan horse represents a valuable reservoir of historical genetic diversity (Kavar and Dovč 2008). More recently, mtDNA variation has also been analysed in the Italian Lipizzan population, linking specific haplotypes with classical mare family lines (Crisà et al. 2024).

Studies of paternal lineages have focused on variation in the male‐specific region of the Y chromosome (MSY). Analyses of Y chromosome haplotypes have shown that Lipizzan horses belong to haplotype group G within the so‐called “crown” group of modern horse lineages (Wallner et al. 2017; Bozlak et al. 2023).

Genetic diversity and inbreeding in Lipizzan horses have also been studied using pedigree information and microsatellite markers (Zechner et al. 2002; Curik et al. 2003; Achmann et al. 2004). More recently, genomic approaches based on high‐density SNP arrays have enabled genome‐wide analyses of population structure, genetic diversity and signatures of selection in Lipizzan horses (Grilz‐Seger, Druml, et al. 2019; Gmel et al. 2023). Genome‐wide analyses have also identified loci associated with body conformation traits (Gmel et al. 2023) and with the grey coat colour phenotype, which is a consequence of progressive greying during the first years of life and is associated with a duplication in the STX17 gene (Curik et al. 2013; Grilz‐Seger, Druml, et al. 2019; Grilz‐Seger, Neuditschko, et al. 2019).

Although the history and genetic structure of the Lipizzan horse have been investigated using pedigree, microsatellite and genomic data, a comprehensive genome‐wide characterization of the historically important population from the Lipica Stud integrating pedigree, STR and SNP data has not yet been performed.

In this study, we analysed pedigree records, microsatellite genotypes and SNP array data of Lipizzan horses from the Lipica Stud Farm to (i) characterize genetic diversity and inbreeding in the reference population, (ii) compare pedigree‐, STR‐ and SNP‐based estimates of inbreeding within the same population, and (iii) identify putative genomic regions relevant for the conservation of genetic diversity in the Lipizzan breed.

## 2 Materials and Methods

### 2.1 Sample Collection

Blood samples were collected from 233 Lipizzan horses from Lipica stud (born between 1992 and 2018) which constituted the reference population. The analysed horses were selected according to the availability of biological samples, and complete pedigree, STR and SNP data. In this time period 785 horses were born in Lipica Stud (390 fillies and 395 colts) to 213 breeding mares and 73 breeding stallions. The sample set includes an active breeding and working Lipizzan population in the stud of Lipica in 2023 and includes representatives of all six classical Lipizzan stallion lines (Conversano (48), Neapolitano (29), Favory (34), Siglavy (32), Maestoso (38), Pluto (50)) and 18 mare family lines (Deflorata (22), Almerina (18), Spadiglia (18), Mercurio (16), Rebecca (16), Presciana (15), Africa (14), Argentina (14), Sardinia (14), Theodorosta (14), Djerbin (12), Englanderia (12), Gidrana (11), Europa (10), Stornella (9), Munja (7), Famosa (3) and Capriola (1)).

### 2.2 DNA Extraction and SNP Array Genotyping

DNA was extracted from 200 μL of peripheral blood using the E.Z.N.A. Tissue DNA Kit (Omega BIO‐TEK, Norcross, GA, USA). Genotyping was performed by Neogen using the GGP Equine 70k BeadChip (Neogen/Illumina, Lincoln, NE, USA) containing 71 607 markers (65 090 autosomal, 1187 mtDNA, 3417 X‐chromosome, and 170 Y‐chromosome markers). The array also includes SNPs used for parentage verification, markers associated with known Mendelian traits in horses, and duplicated SNPs for quality control.

### 2.3 Pedigree Data

The pedigree information of the reference population was extracted from the Slovenian Central Register of Equine Animals. The reference population consisted of the 233 genotyped Lipizzan horses used for comparisons between pedigree, STR and SNP‐based analyses. The pedigree was tested for pedigree loops, duplicated entries, and conflicts between the stated sex and the sex of the animal's parent. Based on additional data from Austrian and Croatian Lipizzan pedigrees, incorrect entries were removed or corrected. The pedigrees of genotyped horses (233 animals) were traced back to the horses that are considered the founders of the Lipizzan breed. After editing, the pedigree comprised 2911 animals, 447 of which were founders, i.e., animals whose parents could not be found in the pedigree (Maignel et al. 1996). In the base population, there were 464 horses with at least one unknown parent (phantom founder).

### 2.4 Pedigree Analysis

The completeness of the pedigree (MacCluer et al. 1983), the inbreeding coefficients (*F*) based on identity by descent (IBD; (Wright 1931)), computed according to (Meuwissen and Luo 1992) and generation interval (*G*_*i*_) as the average age of the parents at the birth of their progeny kept for reproduction (James 1977), were calculated using ENDOG 4.8 software (Gutierrez and Goyache 2005). The expected genetic contributions of founders and the marginal genetic contributions of ancestors to the gene pool were estimated using ENDOG 4.8. Ancestors were ranked using the sequential procedure of Boichard et al. (1997), in which the marginal contribution of an ancestor represents the proportion of the gene pool not already explained by previously selected ancestors. The individual increase of inbreeding (Δ*F*) was calculated and used to estimate effective population size $\left(N_{e}=\frac{1}{2\Delta F}\right)$ following (Goyache et al. 2003). The brief structure of the pedigree, the distribution of *F*, and the longest ancestral path (LAP) were calculated using CFC 1.0 software (Sargolzaei et al. 2006). For dividing inbreeding into ancestral and new inbreeding GRain software v. 2.2 (Baumung et al. 2015; Doekes et al. 2020) was applied. The kinship coefficient was calculated using *kinship2* library (Sinnwell et al. 2014) for R (R Core Team 2024) to detect possible pedigree errors.

**FIGURE 1:**
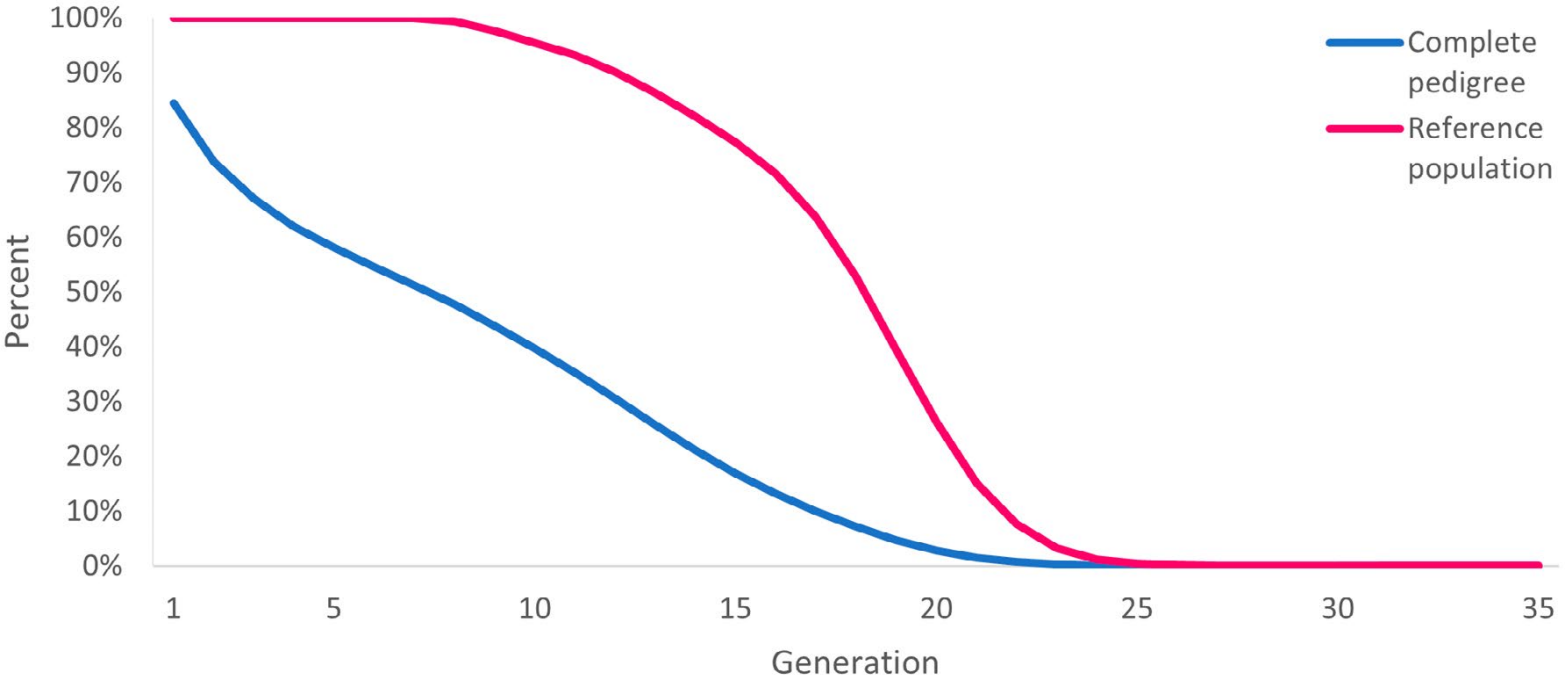
Pedigree completeness by generations in the complete pedigree and in the reference population, used in the analysis, up to the maximal number of traced generations.

In the reference population, the completeness of the pedigree was 100% up to the 7th generation, in the 13th generation it drops below 90%, in the 19th generation below 50%, and in the 25th generation the completeness of the pedigree was less than 1% (Figure 1). Of all horses in the pedigree, the parents are known for 84.5%, of the horses, in the 7th generation we know more than 51% of the ancestors, and in the 22nd generation the ratio of known parents drops below 1%.

### 2.5 Microsatellite Data Analysis

A total of 233 samples were included in the STR analysis. Some older samples were not genotyped for all loci, resulting in approximately 20% missing genotypes for the markers ASB17, ASB23, CA425, HTG7 and HTG10. Genotyping was routinely performed for annual parentage testing, required by the studbook. ISAG STR markers were genotyped using the Equine Genotypes Panel 1.1 Kit (Thermo Scientific). PCR products were analyzed using GeneScan 500 LIZ dye Size Standard (Applied Biosystems) on Applied Biosystems 3500 Series Genetic Analyzer. Fragment analysis was performed using GeneMapper v4.1 software (Applied Biosystems).

The possible presence of null alleles and genotyping errors were analyzed using the Micro‐Checker, v2.2.3 software (Van Oosterhout et al. 2004). The suspect data were omitted from the analysis, no null alleles were identified. GenAlEx (Peakall and Smouse 2012) was used for identification of missing genotypes for each locus, sample size (*N*), number of different alleles (*N*_a_), number of effective alleles (*N*_e_), information index (*I*), observed (*H*_o_), expected (*H*_e_), unbiased expected heterozygosity (*uH*_e_), and fixation index (*F*_IS_). Polymorphic Information Content (*PIC*) was calculated using Cervus 3.0.7 (Kalinowski et al. 2007).

Nei's genetic distances between individuals were calculated using the *poppr* library version 2.9.6 (Kamvar et al. 2014) in R based on a subset of 11 STR loci with call rates above 80%. Markers with higher proportions of missing genotypes were excluded from this analysis to reduce potential bias in distance estimation.

Individual inbreeding coefficients based on microsatellite (STR) markers (*F*_STR_) were estimated in R using the function implemented in the *adegenet* package (Jombart and Ahmed 2011). This function estimates individual inbreeding coefficients using a likelihood‐based approach from multilocus genotype data. The distribution of inbreeding coefficients was plotted using the *ggplot2* library (Wickham 2016) for R.

### 2.6 Quality Control of SNP Array Data

Data quality control was performed using SNP & Variation Suite v8.8.3 (Golden Helix Inc., Bozeman, MT, www.goldenhelix.com). All samples passed the 95% call rate threshold; therefore, no samples were removed. No duplicated samples were detected. SNPs with a call rate less than 95% were removed (70 896 of 71 607 markers remained). After removal of MSY, X chromosome, mtDNA and SNPs with unknown location, 64 626 SNPs remained. The genomic coordinates of the SNPs were based on the equine assembly EquCab3.0. Following the removal of indels, 64 604 SNPs remained. No minor allele frequency filter was applied, because the analyses focused primarily on runs of homozygosity and population diversity parameters. Plink 1.9 (Purcell et al. 2007) and vcftools (Danecek et al. 2011) were used for file format conversion and general SNP data handling. Plink 1.9 was also used to remove duplicated SNPs, resulting in a final dataset of 64 102 SNPs used for all subsequent analyses.

### 2.7 Genetic Diversity Parameters and Effective Population Size Inferred From SNP Data

Population genetic statistics were calculated using the Stacks Populations module (Catchen et al. 2013). The effective population size (*N*_e_) was estimated by the heterozygote excess method of Pudovkin (Zhdanova and Pudovkin 2008) as implemented in NeEstimator V2.1 (Do et al. 2014). To investigate historical effective population size (*N*_e_) and possible demographic bottlenecks, GONE was used, which estimates past Ne from genome‐wide SNP data based on patterns of linkage disequilibrium between markers (Santiago et al. 2020). Only estimates for the most recent 40 generations were presented and interpreted, corresponding to approximately 467 years based on the mean generation interval of 11.68 years.

The genetic kinship coefficient was calculated using Plink 2.0 (Chang et al. 2015) with the ‐horse and ‐make‐king‐table options to obtain pairwise genomic kinship estimates.

### 2.8 Comparison of Inbreeding Estimates From Pedigree, Microsatellite and SNP Data

Inbreeding coefficients were estimated using three complementary approaches. The pedigree‐based inbreeding coefficient (*F*_PED_) represents the probability that two alleles at a locus are identical by descent based on recorded ancestry. Microsatellite‐based inbreeding (*F*_STR_) was estimated from multilocus STR genotype data and reflects deviations from Hardy–Weinberg equilibrium across loci. Genomic inbreeding (*F*_ROH_) was calculated as the proportion of the autosomal genome covered by runs of homozygosity (ROH) detected from genome‐wide SNP data. These approaches capture different aspects of inbreeding and therefore may produce different estimates.

Comparison of inbreeding coefficients was performed using Pearson correlation and visualized using the *ggpubr* library (Kassambara 2026 for R). A 3D scatter plot was generated using the *plotly* library (Sievert 2020) for R.

### 2.9 Analysis of Runs of Homozygosity

Runs of homozygosity (ROH) are most likely the result of the mating between related individuals and represent alleles identical by descent (IBD). The portion of the genome, covered by ROH, provides information about inbreeding and when matings among related individuals occurred (Curik et al. 2014). ROH were identified using SNP and Variation Suite v8.9.0 (Golden Helix Inc., Bozeman, MT, USA). The minimum run length was set to 500 kb, the minimum number of homozygous SNPs within a run was 25, with no heterozygous SNPs allowed, and 5 SNPs with a missing genotype were allowed. ROH were grouped by length: short (to 2.0 Mb), medium (2.0 to 4.0 Mb, 4.0 to 8.0 Mb), and long (8.0 to 16.0 Mb, longer than 16.0 Mb). ROH length categories were defined following approaches used in previous horse genomic studies (Szmatoła et al. 2022). Genomic inbreeding coefficients (*F*_ROH_) were defined as the proportion of the autosomal genome covered by ROH. *F*_ROH_ values were summarized according to stallion lines and mare families to explore variation in genomic inbreeding among major pedigree groups within the Lipica population. The autosomal genome length was set to 2.28 Gb (without unplaced scaffolds).

### 2.10 Selection Signatures

Selection signatures were analyzed using ROH islands and by the integrated haplotype score (iHS) method. For the ROH‐island analysis, the proportion of horses in which each SNP occurred within a ROH was calculated. SNPs in the upper 1% of the distribution of ROH occurrence were considered candidate ROH‐island SNPs. In the present dataset, this threshold corresponded to SNPs occurring within a ROH in at least 104 of the 233 horses (44.6% of the reference population).

For the iHS analysis, genotypes were phased chromosome‐wise using Beagle 5.4 (Browning et al. 2021). The analysis was performed in R using the rehh package version 3.2.2. Integrated haplotype homozygosity statistics were calculated for each chromosome using the scan_hh function with default parameters (limhaplo = 2, limhomohaplo = 2, limehh = 0.05, limehhs = 0.05, phased = TRUE, polarized = FALSE, no gap scaling or maximum‐gap restriction, and linear interpolation of EHH values). Standardized iHS values were subsequently calculated using the ihh2ihs function with default parameters, including a frequency‐bin width of 0.025, a minimum minor allele frequency of 0.05, and within‐bin standardization. SNPs with absolute standardized iHS values greater than 3 (|iHS| > 3) were considered candidate selection signals.

Candidate genomic regions identified using either the ROH‐island or iHS approach were annotated using Ensembl release 111 based on the EquCab3.0 assembly. For ROH islands, genes located within the identified regions were retrieved, whereas for significant iHS signals, genes directly overlapping the candidate SNPs and genes located within ±500 kb were recorded. The identified genes were compared with candidate genes previously reported in horse studies. Genes associated with relevant biological functions in other mammalian species were additionally recorded for exploratory comparison using information retrieved from the OMIA database (Tammen et al. 2024). The genomic distribution of ROH occurrence was visualized using the qqman package in R, whereas iHS results were visualized using the manhattanplot function in the rehh package (Gautier and Vitalis 2012; Klassmann and Gautier 2022).

### 2.11 Functional Enrichment Analysis

Functional enrichment analysis of genes located within ROH islands was performed using the PANTHER classification system (Mi and Thomas 2009). The list of annotated genes was submitted to the PANTHER Overrepresentation Test to identify significantly enriched biological pathways. The analysis was conducted using *Equus caballus* as the reference genome and the PANTHER Protein class database. Statistical significance was evaluated using Fisher's exact test with false discovery rate (FDR) correction.

## 3 Results

### 3.1 Pedigree Analysis

After integration and editing of the pedigree, the longest ancestral path bridged 35 generations, with an average of 7.53 discrete generation equivalents for the whole pedigree and 17.03 generations for the genotyped horses. The mean inbreeding coefficient for all animals included in the analysis (*n* = 2911), for inbred animals (*n* = 1807), and for the reference population was, 4.95%, 7.97% and 11.55%, respectively (Table 1). The maximum inbreeding coefficient was 36.85%, in the reference population 21.72%, the average relatedness for all animals was 6.37%, and for the reference population 12.80%. The ancestral inbreeding coefficients calculated for the members of the reference population according to (Ballou 1997) vary between 44.23% and 62.06%, while the ancestral inbreeding coefficients according to (Kalinowski et al. 2000) vary between 5.93% and 15.42%. The Kalinowski new inbreeding coefficients range from 0.81% to 6.3% with an average of 1.92%.

**TABLE 1:** Pedigree‐based inbreeding coefficients calculated using pedigree depths of 2, 5, 10, and all available generations. Results are reported separately for all animals included in the edited pedigree (*n* = 2911) and for the genotyped reference population from the Lipica Stud Farm (*n* = 233).

Generations	Pedigree‐based inbreeding coefficient (%) All animals in the edited pedigree (*n* = 2911)			Pedigree‐based inbreeding coefficient (%) Genotyped reference population (*n* = 233)		
	Mean	Min	Max	Mean	Min	Max
2	0.15	0.00	25.00	0.05	0.00	12.50
5	1.60	0.00	31.03	1.96	0.00	12.50
10	3.55	0.00	34.40	5.10	0.93	16.07
ALL	4.95	0.00	36.85	11.55	6.76	21.72

The mean maximum number of generations, the mean complete number of generations and the mean generation equivalent for the entire pedigree were 14.84, 3.36 and 7.53, respectively. For the reference population, these values were higher (31.55, 7.87 and 17.03, respectively), reflecting the deeper pedigree information available for the genotyped horses included in the reference population. We detected some high inbred matings between half‐siblings (22) and seven matings between parents and offspring. The effective population size, calculated using the individual increase of inbreeding in the reference population, was 69.51.

The depth of the pedigree across generations is presented in Figure 1. The mean generation interval was 11.68 ± 0.08 for all animals and 14.36 ± 0.49 for the reference population (Table 2).

**TABLE 2:** Generation interval.

	All animals				Reference population			
	*n*	Interval	SD	SE	*n*	Interval	SD	SE
Father‐son	702	12.93	7.51	±0.28	10	18.10	4.36	±1.38
Father‐daughter	1579	12.40	5.77	±0.15	44	16.36	5.18	±1.64
Mother‐son	697	10.64	5.01	±0.19	10	12.40	3.34	±1.06
Mother‐daughter	1582	10.88	4.76	±0.12	44	11.95	4.30	±1.36
Total	4560	11.68	5.72	±0.08	108	14.36	5.14	±0.49

When extracting just sampled animals with their progeny, there were 54 breeding animals (44 mares and 10 stallions) having between one and 18 foals with an average of 3.39 foals per horse. The pedigree depth did not differ between the analysed mares and stallions (Figure S1). In the whole pedigree, in the 5th generation on the mare side, there are 52.0% of known ancestors, and on the stallion side, 61.7%.

When analyzing all animals, 13 ancestors explained 50.4% of the gene pool (Table S1), whereas in the reference population, only 6 ancestors (Table 3) explained 50.8% of the gene pool. A total of 423 ancestors had a non‐zero marginal genetic contribution to the gene pool of the complete pedigree population, whereas 68 ancestors had a non‐zero marginal contribution to the gene pool of the reference population. In the total population, 464 founders (including phantom founders) were identified.

**TABLE 3:** Marginal genetic contributions of the six highest‐ranked ancestors, which cumulatively explained 50.8% of the gene pool of the reference population comprising 233 genotyped Lipizzan horses. All horses in the table are males.

Ancestor	Year of birth	Country of birth	Sex	Cumulative genetic contribution (%)	Marginal genetic contribution (%)
116 CONVERSANO GAETANA IV	1947	HR	M	11.55	11.55
045 MAESTOSO BONAVOJA 45	1967	SI	M	22.39	10.84
321 FAVORY DUBOVINA IV	1964	SI	M	31.27	8.88
513 NEAPOLITANO BATOSTA XXI	1956	SI	M	38.42	7.15
090 PLUTO NAVARRA 90	1972	SI	M	45.31	6.89
FAVORY RATISBONA II	1829	SI	M	50.81	5.50

**FIGURE 2:**
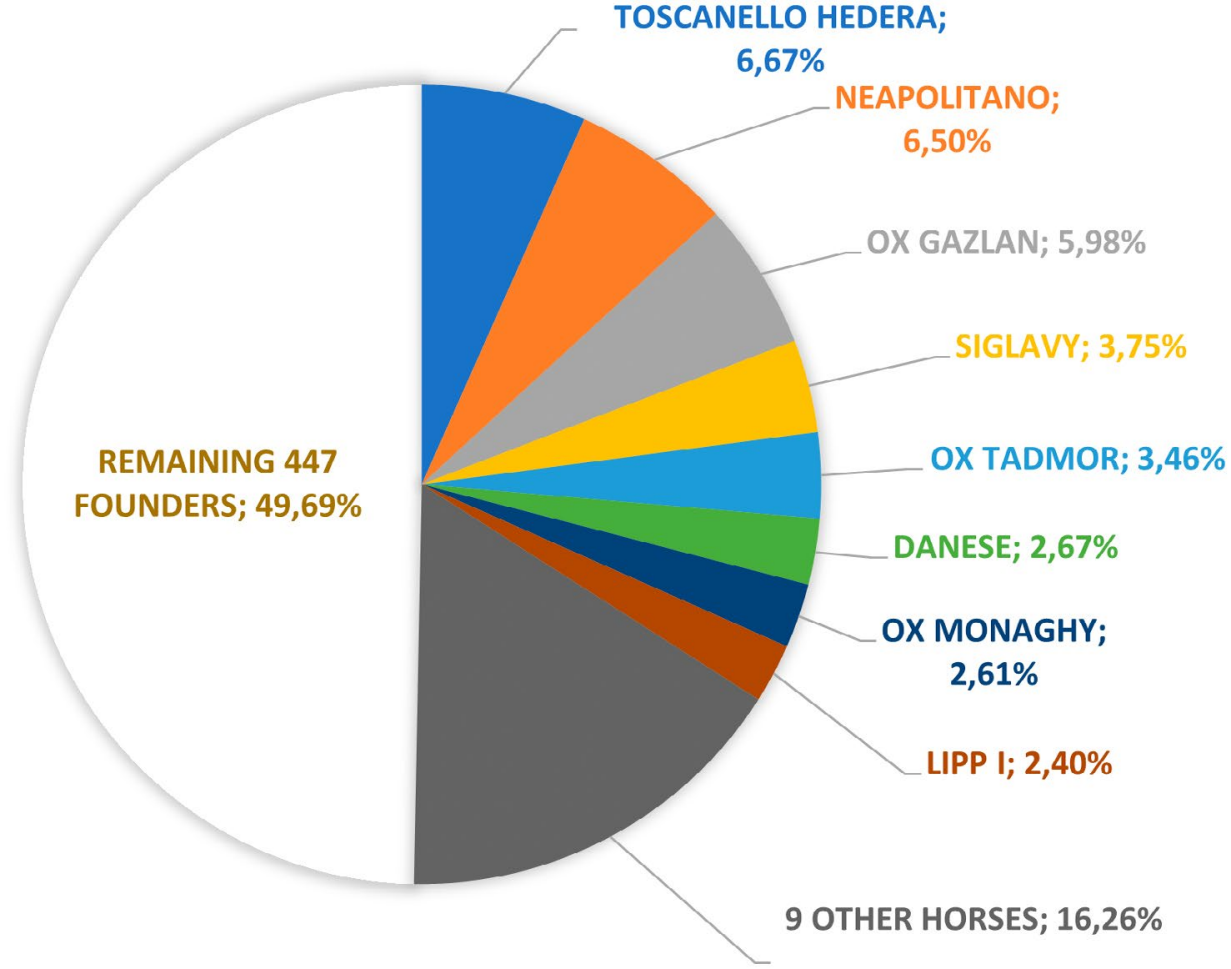
Genetic contributions of the 17 highest‐contributing founders and the remaining 447 founders to the gene pool of the reference population.

The 17 founders with the largest genetic contributions cumulatively accounted for 50.3% of the gene pool of the reference population. Toscanello Hedera had the largest genetic contribution (Figure 2). The first Toscanello stallion originated from Pisa, however, the first representative of this stallion line recorded in the Lipizzan pedigrees, Toscanello Rappe, was born in 1771 in Kladrub. Mating this stallion with the Karst mare Hedera produced Toscanello Hedera, also known as Toscanello I, Hermelin, which was widely used for breeding in Lipica until 1806 (Grilz‐Seger and Druml 2011).

### 3.2 Microsatellite Data Analysis

The microsatellite data contained genotypes for 17 loci from the ISAG Equine comparison test set (*AHT4, AHT5, ASB2, ASB17, ASB23, HMS2, HMS3, HMS6, HMS7, HTG4, HTG10, VHL20, CA425, HMS1, HTG6, HTG7* and *LEX3*). The proportion of missing genotypes per marker is presented in Table 4. Due to its location on the chromosome X, we excluded the marker *LEX3* from further analysis. The average observed heterozygosity (*H*_o_) for all markers was 0.68, the average number of alleles was 5.9 per locus and number of effective alleles was 3.2. The average Wright's fixation index (*F*_IS_) was −0.02 (Table 5).

**TABLE 4:** Missing data per microsatellite.

	*N* valid	*N* missing	% Missing
*AHT4*	233	0	0.00
*AHT5*	215	18	7.73
*ASB2*	227	6	2.58
*ASB17*	186	47	20.17
*ASB23*	186	47	20.17
*CA425*	185	48	20.60
*HMS1*	214	19	8.15
*HMS2*	231	2	0.86
*HMS3*	233	0	0.00
*HMS6*	232	1	0.43
*HMS7*	231	2	0.86
*HTG4*	232	1	0.43
*HTG6*	229	4	1.72
*HTG7*	186	47	20.17
*HTG10*	185	48	20.60
*VHL20*	233	0	0.00

**TABLE 5:** Microsatellite loci statistics. Sample size (*N*), number of alleles (*N*_a_), number of effective alleles (*N*_e_), information index (*I*), observed heterozygosity (*H*_o_), expected heterozygosity (*H*_e_), unbiased expected heterozygosity (*uH*_e_), fixation index (*F*_IS_) and polymorphism information content (*PIC*) for 16 STR markers in the Lipizzan population.

Loci	*N*	*N*_a_	*N*_e_	*I*	*H*_o_	*H*_e_	*uH*_e_	*F*_IS_	*PIC*
*AHT4*	233	6.00	3.93	1.50	0.81	0.75	0.75	−0.08	0.70
*AHT5*	215	4.00	3.50	1.31	0.76	0.72	0.72	−0.07	0.66
*ASB2*	227	6.00	3.20	1.37	0.70	0.69	0.69	−0.01	0.64
*ASB17*	186	7.00	3.98	1.54	0.80	0.75	0.75	−0.07	0.71
*ASB23*	186	8.00	5.57	1.81	0.86	0.82	0.82	−0.04	0.79
*CA425*	185	6.00	1.80	0.87	0.44	0.45	0.45	0.00	0.41
*HMS1*	214	4.00	2.28	0.92	0.54	0.56	0.56	0.04	0.46
*HMS2*	231	7.00	3.19	1.31	0.71	0.69	0.69	−0.04	0.64
*HMS3*	233	6.00	3.99	1.46	0.69	0.75	0.75	0.08	0.71
*HMS6*	232	5.00	2.63	1.14	0.63	0.62	0.62	−0.01	0.55
*HMS7*	231	6.00	3.28	1.39	0.69	0.70	0.70	0.00	0.65
*HTG4*	232	6.00	2.25	1.03	0.57	0.56	0.56	−0.02	0.48
*HTG6*	229	5.00	2.77	1.28	0.65	0.64	0.64	−0.01	0.60
*HTG7*	186	3.00	2.65	1.04	0.62	0.62	0.62	0.00	0.55
*HTG10*	185	9.00	3.14	1.39	0.68	0.68	0.68	0.01	0.63
*VHL20*	233	7.00	3.73	1.52	0.79	0.73	0.73	−0.08	0.69
Average	214.88	5.94	3.24	1.30	0.68	0.67	0.67	−0.02	0.62
SE	5.29	0.38	0.23	0.06	0.03	0.02	0.02	0.01	0.03

**FIGURE 3:**
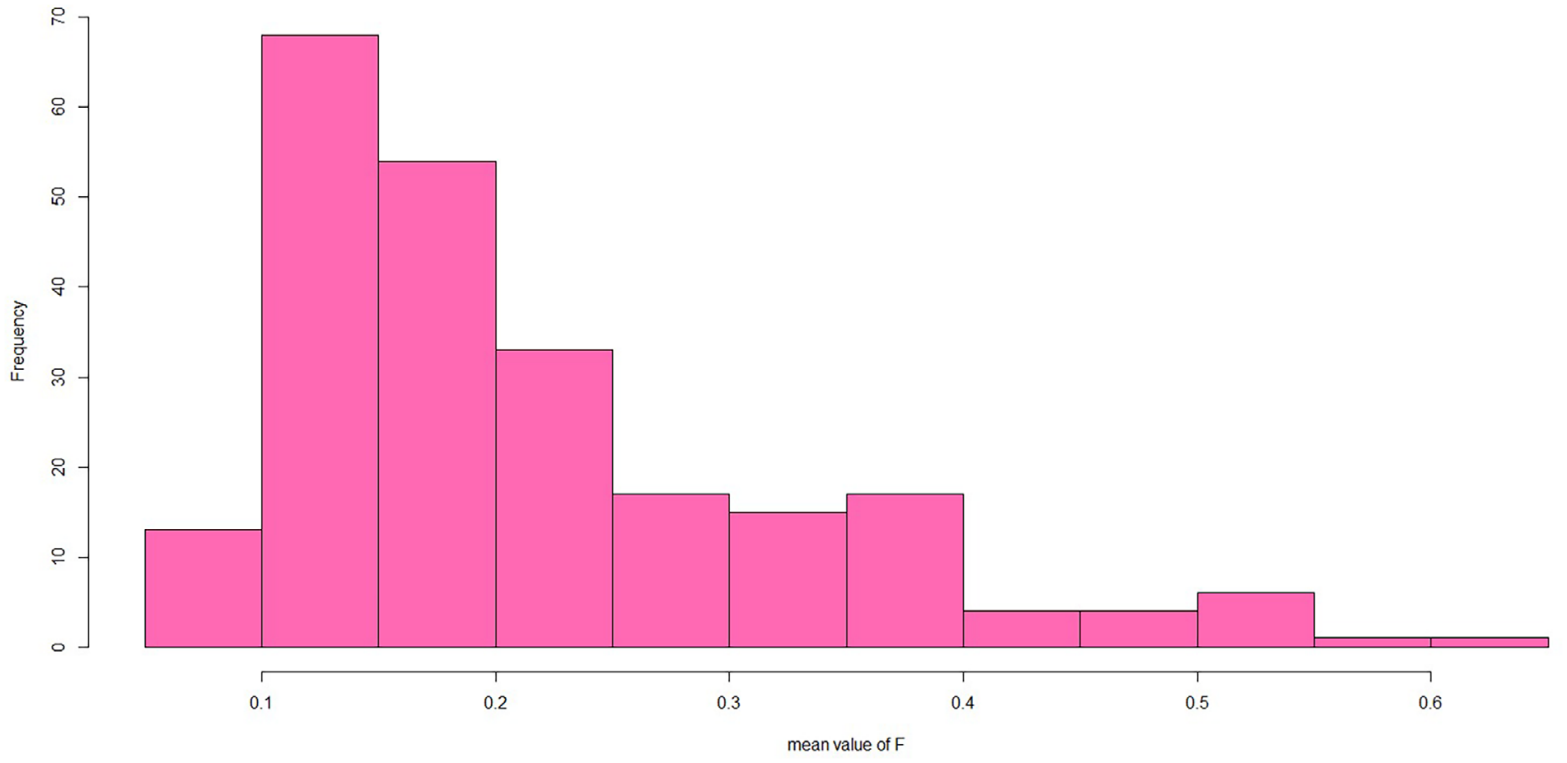
Distribution of mean *F*_STR_ across individuals.

The inbreeding coefficient of individuals based on microsatellite (STR) markers (*F*_STR_) was estimated for all genotyped horses. In 29.18% of the genotyped horses, the *F*_STR_ was between 0.10 and 0.15. The highest inbreeding coefficient based on STR was 0.61 (Figure 3).

Pairwise Nei's genetic distances between individuals did not reveal clear clustering patterns within the population (Figure S2). The largest Nei's distance (2.773) was estimated between 542 SAMIRA XXII and 616 WERA XXXIV. Comparison of their pedigrees revealed that they were both granddaughters of M. BONAVOJA 45, but all other ancestors in their five‐generation pedigrees were different.

### 3.3 Analysis of SNP Array Data

**FIGURE 4:**
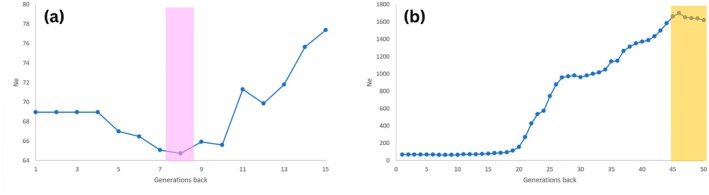
Estimated effective population sizes in past generations with coloured bottlenecks. (a) Time of the Second World War in pink. (b) and (c) breed formation process and establishment of Lipica Stud Farm in yellow.

After quality control, 64 604 autosomal SNPs were retained for downstream analyses. Among these loci, 55 994 were polymorphic in the analysed population. The overall nucleotide diversity (π) was 0.31274, the average major allele frequency (P) was 0.76753 and *F*_IS_ was −0.02046. The effective population size estimated using the heterozygote‐excess method of Pudovkin was 24.9 when all alleles were considered. Changes in effective population size estimated by GONE over the most recent 40 generations are presented in Figure 4. The estimated (Ne) for the most recent generation was 68.95.

### 3.4 Genomic Inbreeding

**FIGURE 5:**
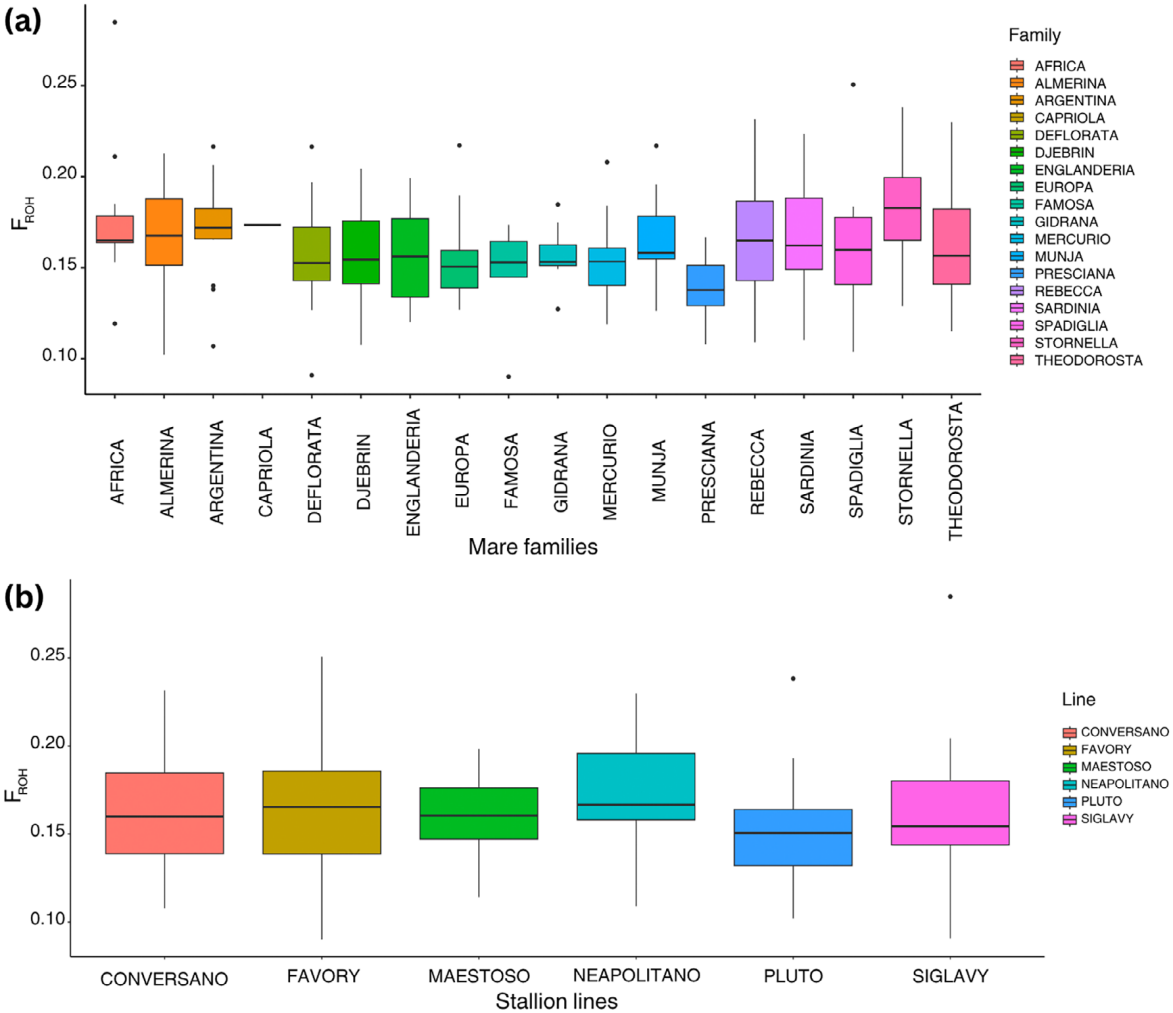
Distribution of *F*_ROH_ according to (a) mare families and (b) stallion lines in the Lipica Stud Farm reference population.

The genomic inbreeding estimated from ROH ranged between 9.01% and 28.49% with an average of 16.09%. The average length of all ROH was 2.990 Mb, with the average number of ROH longer than 1 Mb of 97 per sample, resulting in 367.05 Mb of the genome covered by ROH. The average total length of short ROH (1–2 Mb) per sample (63.264 Mb) was comparable with the sum length of the longest ROH (> 16 Mb; 63.899 Mb) as presented in Table S2. In horses from Lipica, we found the highest portions of ROH with lengths between 4 and 8 Mb (Figure S3), which likely reflects common ancestry approximately 5–10 generations back and indicates a bottleneck. Only minor differences in *F*_ROH_ among mare families and stallion lines were observed (Figure 5).

### 3.5 Comparison of Inbreeding Coefficients

**FIGURE 6:**
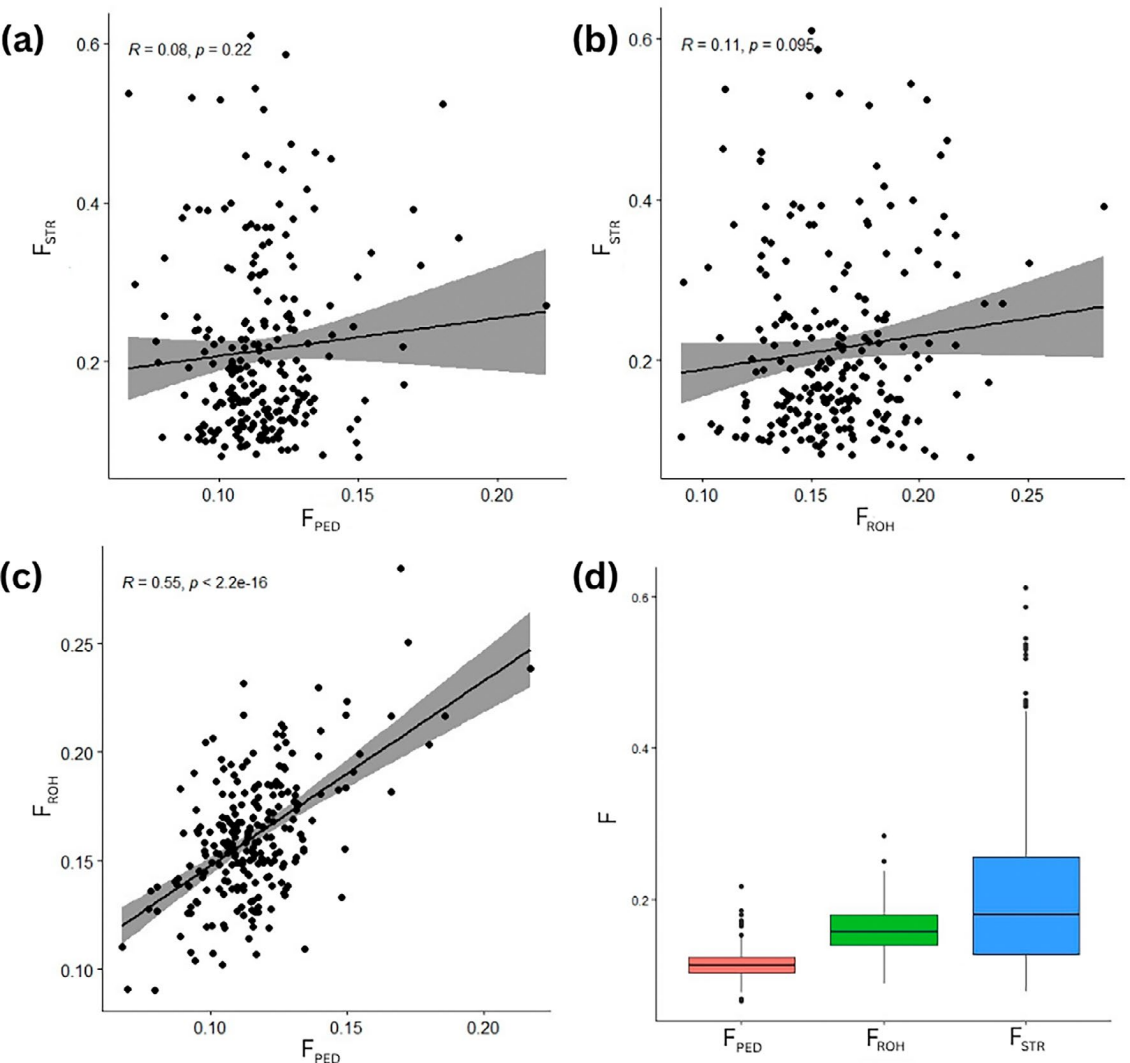
Comparison of inbreeding coefficients. (a) Correlation between *F*_PED_ and *F*_STR_ was not significant. (b) Correlation between *F*_ROH_ and *F*_STR_ was not significant. (c) Correlation between *F*_PED_ and *F*_ROH_ was moderately positive. (d) Boxplots of estimated inbreeding levels for *F*_PED_ with the lowest values, *F*_ROH_ with intermediate values and the *F*_STR_ with the highest values of *F*.

We compared inbreeding coefficients from pedigree and SNP data and used LAP as a third variable (Figure S4). When comparing inbreeding coefficients calculated from pedigree data, STR and ROH, we got a moderate positive correlation between *F*_PED_ and *F*_ROH_, whereas the other two correlations were not significant (Figures 6 and S4).

The lowest inbreeding values were estimated from pedigree data with a minimum of 0.067 and a maximum of 0.217. The highest maximum value was estimated from STR data and reached 0.61 for 573 SIGLAVY GRATIOSA III. The average values of the estimated inbreeding levels for *F*_PED_, *F*_STR_ and *F*_ROH_ were 0.12, 0.21, and 0.16, respectively (Table 6). Correlation between Kalinowski new inbreeding coefficient and ROH‐based inbreeding coefficient, calculated from ROH islands longer than 16 Mbp, is moderately positive (Figure S5).

**TABLE 6:** Inbreeding coefficient estimations based on different data sets.

	LAP	*F*_PED_	*F*_STR_	*F*_ROH_	*F*_A_Bal_	*F*_A_Kal_	*F*_N_Kal_	*F*_ROH16_
Min	28	0.067	0.079	0.090	0.442	0.059	0.008	0.007
Max	35	0.217	0.611	0.285	0.621	0.154	0.063	0.127
Average	31.55	0.116	0.214	0.161	0.527	0.096	0.019	0.028

Abbreviations: *F*_A_Bal_, ancestral inbreeding according to Ballou; *F*_A_Kal_, ancestral inbreeding according to Kalinowski; *F*_N_Kal_, new inbreeding according to Kalinowski; *F*_PED_, pedigree based inbreeding; *F*_ROH_, ROH based inbreeding; *F*_ROH16_, ROH based inbreeding for islands longer than 16 Mbp; *F*_STR_, microsatellites‐based inbreeding; LAP, longest ancestral path.

### 3.6 Selection Signatures

**FIGURE 7:**
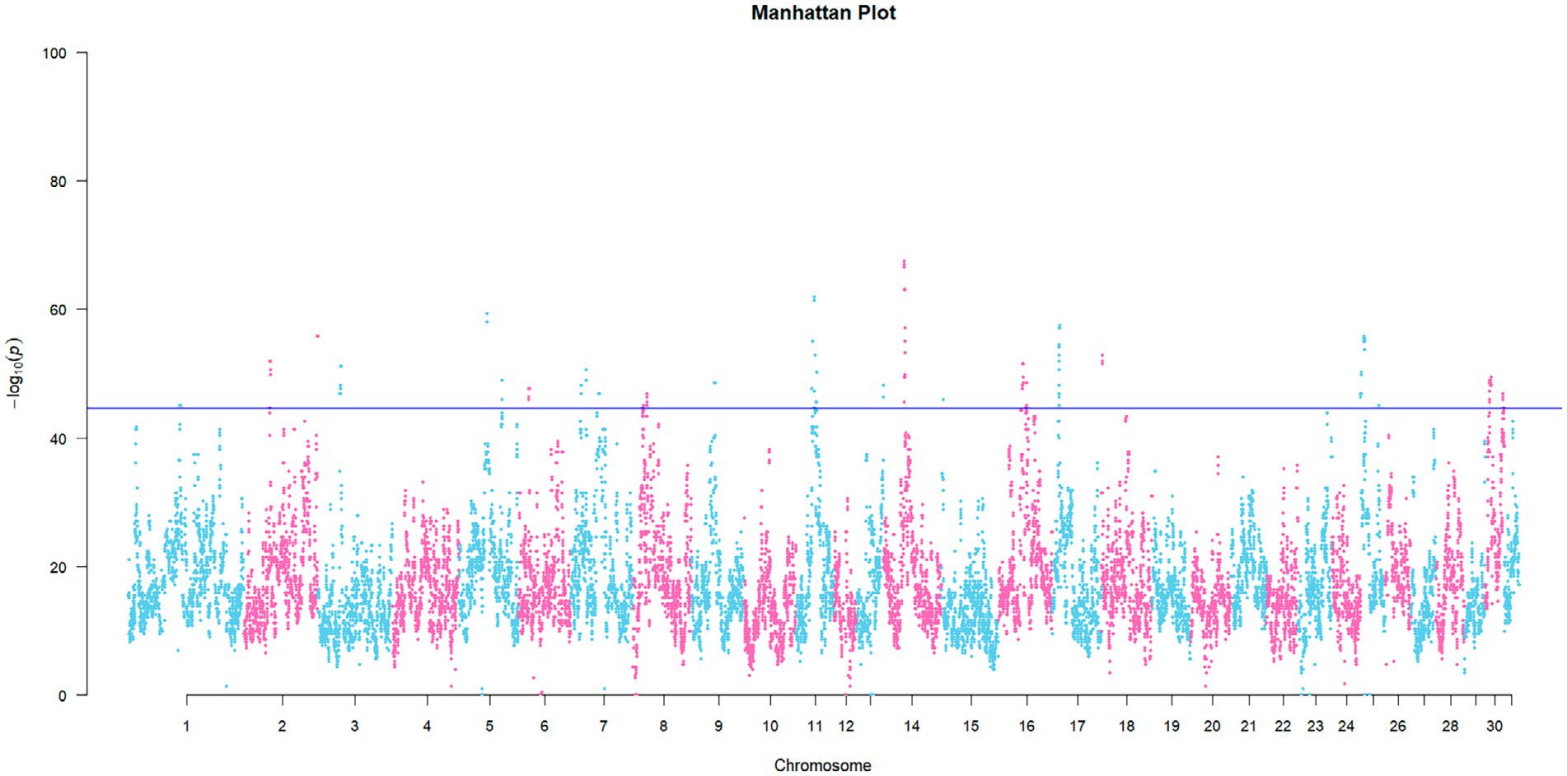
Proportion of overlapping ROH segments shared among the analyzed Lipizzan horses.

Application of the ROH‐island criterion identified 646 candidate SNPs (Figure 7). The corresponding genomic regions were annotated using Ensembl BioMart, release 111, based on the EquCab3.0 assembly, and included 354 protein‐coding genes. The complete list of ROH‐island regions and their annotations is provided in Table S3. Among these regions, we identified 24 candidate genes that have previously been associated with biological functions in horses (Table 7). These genes were detected in the present Lipica dataset, whereas the reported phenotype associations originate either from Lipizzan horses or from other horse breeds, including breeds historically contributing to the Lipizzan gene pool. Additional candidate genes located within ROH islands and previously associated with biological functions or traits in other mammalian species are presented in Table S4.

**TABLE 7:** Candidate genes located within ROH islands identified in the Lipica Stud population and previously associated with biological functions or traits in Lipizzan or other horse breeds.

Chromosome	Gene	Reported phenotype/function	Breed(s)	References
1	*GRID1*	Pubertal timing	Thoroughbred	Asadollahpour Nanaei et al. (2020)
2	*PER3*	Circadian regulation of locomotor activity and skeletal muscle gene expression	Various lightweight breeds	Martin et al. (2010)
3	*MC1R*	Coat colour extension, red pigment	Standardbred, Thoroughbred, Swedish Warmblood, North Swedish horse, Belgian, Arabian, American Paint Horse, Icelandic horse, Welsh pony, Shetland pony, New Forest pony, Gotland pony	Marklund et al. (1996)
5	*PRKAB2*	Energy metabolism and skeletal muscle energy homeostasis (AMPK pathway)	Arabian	Szcześniak et al. (2016)
8	*PEBP1*	Male fertility	Horse	Griffin et al. (2022)
8	*TMEM120B*	Fat cell differentiation	Quarter Horse racing line	Santos et al. (2023)
8	*CFAP251*	Sperm motility	Quarter Horse racing line	Santos et al. (2023)
11	*STXBP4*	Environmental adaptation	Noriker	Grilz‐Seger, Neuditschko, et al. (2019)
11	*NOG*	Skeletal muscle development	Polish Coldblood	Szmatoła et al. (2022)
11	*HSF5*	Oocyte development/female fertility	Quarter Horse‐type mares	Cox et al. (2015)
11	*TEX14*	Male reproductive function	German warmblood breeds: Hanoverian, Holsteiner, Oldenburg, Trakehner	Nolte et al. (2019)
11	*NXPH3*	Locomotion/endurance traits	Soviet heavyweight	Dementieva et al. (2023)
13	*AXIN1*	Osteochondrosis susceptibility	Standardbred, Warmblood	Kinsley et al. (2015)
14	*ARHGAP26*	Skeletal muscle response to exercise	Thoroughbred	Han, McGivney, et al. (2020)
16	*CTNNB1*	Wound repair/exuberant granulation tissue	Standardbred mares	Miragliotta et al. (2008)
16	*RPL14*	Equine behaviour/coping with early training/stress adaptation	Thoroughbred	Holtby et al. (2023)
17	*RXFP2*	Reproductive physiology; relaxin signalling and placental function	Horse	Holtby et al. (2023)
25	*STX17*	Grey coat colour and melanoma susceptibility	Lipizzan, Andalusian, Arabian, Thoroughbred, Welsh Pony, Icelandic Horse, Quarter Horse, Miniature Horse, Shetland Pony, Connemara Pony	Rosengren Pielberg et al. (2008)
25	*RUSC2*	Joint‐specific osteochondrosis‐related gene expression in leukocytes	Belgian Warmblood	Mendoza et al. (2015)
25	*NR4A3*	Grey coat colour and melanoma susceptibility	Lipizzan, Andalusian, Arabian, Thoroughbred, Welsh Pony, Icelandic Horse, Quarter Horse, Miniature Horse, Shetland Pony and Connemara Pony	Switonski et al. (2017)
25	*NR5A1*	Steroidogenesis/testicular function	Quarter Horse	Valdez et al. (2019)
25	*NR6A1*	Cryptorchidism/testicular function/male fertility	Guanzhong, Chakouyi	Han, Chen, et al. (2020)
30	*ENAH*	Exercise‐related traits/athletic performance	Mongolian horse: Abaga and Wushen	Pan et al. (2022)
30	*CACNA1S*	Recurrent exertional rhabdomyolysis (RER)/muscle calcium regulation	Thoroughbred	Dranchak et al. (2006)

Functional enrichment analysis of genes located within ROH islands and already associated with phenotypic traits in horses was performed using the PANTHER classification system. The analysis revealed a significant enrichment of the gonadotropin‐releasing hormone (GnRH) receptor signalling pathway. Three genes (*NR5A1*, *CACNA1S* and *PRKAB2*) from the identified ROH regions were associated with this pathway. No other pathways remained significant after false discovery rate correction.

**FIGURE 8:**
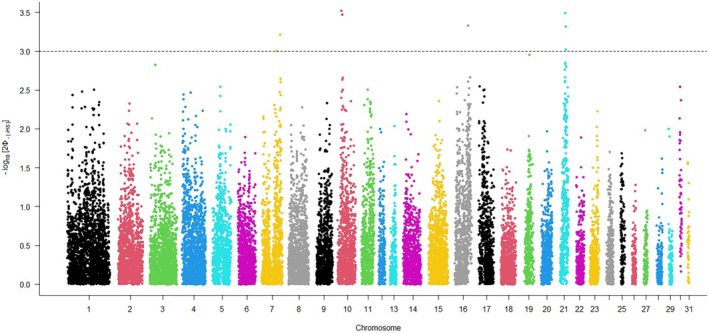
The integrated haplotype score (iHS) indicates within‐breed signatures of selection. Signals exceeding the threshold (|iHS| > 3) were detected on four chromosomes: ECA7 (*TENM4*, *DENND5A*), ECA10 (*ZNF114*, *ENSECAG00000006255*), ECA16 (lncRNA), and ECA21 (no overlapping protein‐coding genes; nearby genes include *NPR3*, *SUB1*, *TARS1*, and *ZFR*).

Results from the iHS analysis identified signals exceeding the threshold value of 3.0 across four chromosomes (ECA7, ECA10, ECA16, and ECA21) (Figure 8). Annotation of these positions identified protein‐coding genes directly overlapping the significant signals: *ZNF114*, *TENM4*, *DENND5A*, and *ENSECAG00000006255* (predicted ortholog to the human *FPR2* gene). *TENM4* has previously been reported among candidate genes expressed in equine tendon tissue (Kuemmerle et al. 2016).

Within the ±500 kb window, additional protein‐coding genes were identified in proximity to significant loci, including *NPR3*, *SUB1*, *TARS1*, and *ZFR*, alongside multiple long non‐coding RNA (lncRNA) genes. Functional classification of the identified protein‐coding genes suggests involvement in diverse biological processes, including transcriptional regulation (*ZNF114*, *SUB1*), intracellular signaling (*NPR3*), RNA metabolism (ZFR), and protein synthesis (*TARS1*).

## 4 Discussion

Our study provides an integrated characterization of the Lipizzan horse population from the Lipica Stud Farm using pedigree, microsatellite, and SNP data. The analysed dataset includes representatives of all classical stallion lines and mare families currently present in the stud and therefore provides an overview of the genetic structure of the Lipizzan population bred in Lipica. These results complement previous genetic studies conducted in Lipizzan horses from different European studs.

The pedigree‐based inbreeding coefficient estimated for the Lipica reference population in the present study was 11.55%, which is slightly higher than previously reported for Lipica horses by Zechner et al. (2002), who estimated an inbreeding coefficient of 10.73% for Lipica and 10.81% across all analysed Lipizzan studs. Curik et al. (2003) also reported somewhat lower average pedigree‐based inbreeding values for Lipizzan horses from different state studs (10.3%). The higher value observed in the present study may be explained by the greater completeness and depth of the pedigree information used in this study. The reference population consisted primarily of recently sampled animals with relatively complete ancestral records, whereas the complete pedigree dataset also includes older historical animals with incomplete parental information. Such incomplete pedigrees may lead to underestimation of pedigree‐based inbreeding when all animals are analysed together.

Population size and the structure of breeding pedigrees can substantially influence inbreeding levels. For example, a pedigree‐based inbreeding coefficient of 0.19 was recently reported for the Italian Lipizzan horse population in Monterotondo (Crisà et al. 2024). Differences among studies may reflect not only breeding practices but also differences in pedigree depth and completeness, which affect the accuracy of pedigree‐based inbreeding estimates. The relatively deep and complete pedigree of the reference population supports a robust evaluation of pedigree‐based inbreeding and ancestor contributions, although incomplete information in the earliest generations may still lead to some underestimation of historical inbreeding.

Analysis of ancestor contributions showed that the ten most influential ancestors identified in the present study largely correspond to those reported by (Zechner et al. 2002), although minor differences in their relative ranking were observed. In the present dataset, six ancestors explained approximately 50% of the recent gene pool, whereas eight ancestors were required to reach the same proportion in the earlier study. Similarly, 17 founders contributed more than half of the genetic variation in the reference population. These results reflect the historically structured breeding system of the Lipizzan horse and the uneven genetic contributions of particular founder lines.

The microsatellite and ROH results reflect different aspects of genetic diversity and inbreeding and are therefore not contradictory. An *F*_IS_ value close to zero indicates that the observed heterozygosity at the STR loci was similar to that expected from the allele frequencies within the sampled population; it does not indicate an absence of historical inbreeding or genome‐wide autozygosity. Furthermore, the ISAG microsatellite panel comprises a relatively small number of highly polymorphic loci selected primarily for parentage verification and therefore has limited resolution for estimating genome‐wide inbreeding. In contrast, *F*_ROH_ directly measures the proportion of the autosomal genome contained in homozygous segments and thus reflects realised autozygosity resulting from shared ancestry. Moderate microsatellite diversity and *F*_IS_ value close to zero are therefore compatible with the appreciable genomic inbreeding detected by ROH in the Lipica population.

The distribution of ROH length classes suggests contributions from both recent and more distant inbreeding events (Kirin et al. 2010). The relatively high proportion of ROH segments between 4 and 8 Mb suggests common ancestors approximately 5–10 generations back. Considering the average generation interval of approximately 11.7 years in the Lipizzan population, this period broadly corresponds to demographic events affecting the breed during the twentieth century, including population reductions associated with the two world wars (Dovc et al. 2006). The GONE analysis also indicated changes in effective population size during the recent demographic history of the breed, although individual fluctuations could not be conclusively attributed to specific historical events.

ROH detection may be influenced by the methodological criteria used to define homozygous segments, and shorter ROH are generally more likely than longer segments to occur by chance. Theoretical considerations regarding Type I error suggest that more stringent SNP‐number thresholds may reduce the probability of detecting chance homozygosity (Lencz et al. 2007; Meyermans et al. 2020). However, ROH detection in the present study was based on a combination of criteria, including physical segment length, SNP number, the absence of heterozygous calls and the permitted number of missing genotypes. In addition, the shortest ROH class (1–2 Mb) accounted for approximately 17% of the total ROH length, whereas more than 80% was attributable to segments longer than 2 Mb. The potential influence of false‐positive short ROH on the overall FROH estimate is therefore expected to be limited, although results involving the shortest ROH class should be interpreted with caution. ROH islands supported predominantly by short overlapping segments may likewise be more sensitive to the selected detection criteria. Selection signature analyses based on ROH islands and the integrated haplotype score (iHS) revealed several genomic regions that may have been influenced by selection. ROH islands typically reflect genomic regions shaped by long‐term selection or genetic drift, whereas the iHS statistic is designed to detect more recent or ongoing positive selection. In the present study, most candidate regions were identified through the ROH island analysis. Within these regions, several genes, previously associated with biologically relevant traits in horses, were identified.

For example, *GRID1* has previously been associated with pubertal timing in Thoroughbred horses (Asadollahpour Nanaei et al. 2020). Two genes located on ECA8, *PEBP1* and *CFAP251*, are involved in reproductive functions related to sperm maturation and motility (Griffin et al. 2022; Santos et al. 2023). Genes related to coat colour were also detected within ROH islands, including *MC1R*, which determines the extension locus responsible for red pigmentation (Marklund et al. 1996) and *STX17*, associated with the grey coat phenotype characteristic of Lipizzan horses (Seltenhammer et al. 2003). In addition, genes such as *SPRY4* and *NR4A3*, previously linked to melanoma susceptibility in grey horses (Switonski et al. 2017; Grilz‐Seger, Druml, et al. 2019) were located within ROH regions detected in this population. The genes listed in Table 7 were identified within ROH islands detected in the Lipica population analysed in this study, whereas their reported trait associations originate mainly from studies conducted in other horse breeds. Additional candidate genes identified within ROH islands and previously associated with biological functions or traits in other mammalian species are summarized in Table S4.

Functional enrichment analysis indicated a significant overrepresentation of genes involved in the gonadotropin‐releasing hormone (GnRH) receptor signalling pathway. GnRH signalling plays a central role in the hypothalamic–pituitary–gonadal axis and regulates reproductive processes by releasing luteinizing hormone (LH) and follicle‐stimulating hormone (FSH) (Khan et al. 2025). The presence of genes associated with this pathway within ROH islands may therefore suggest genomic regions potentially related to reproductive physiology, although further studies would be required to confirm any functional implications.

The ROH islands may arise from the combined effects of selection, demographic history, and genetic drift, whereas the resolution of haplotype‐based statistics such as iHS depends on marker density. Because the present study relied on a medium‐density SNP array, the detected ROH islands and selection signals should be regarded as putative candidate regions that warrant further investigation using higher‐density SNP arrays or whole‐genome sequence data.

The results of the present study should also be interpreted in light of several limitations. The analysed dataset includes horses born up to 2018 and therefore does not capture the most recent generations of the Lipica population. In addition, the resolution of genomic analyses is constrained by the density of the SNP array and the relatively small set of STR markers used for microsatellite analyses. Future studies incorporating more recent cohorts and higher‐density genomic data may therefore provide additional insights into the ongoing genetic dynamics of the Lipizzan population.

Overall, the results of this study are broadly consistent with the historical development and breeding structure of the Lipizzan horse population described in earlier pedigree‐based studies (Zechner et al. 2002). By combining pedigree, microsatellite, and SNP‐based analyses within the same reference population, the present study provides a complementary perspective on the genetic diversity and genomic structure of the Lipica Stud Farm population and contributes to the understanding and conservation of genetic diversity within the Lipizzan breed.

## Author Contributions

**Tamara Ferme:** data curation, investigation, writing – original draft. **Minja Zorc:** conceptualization, methodology, software, data curation, validation, supervision, writing – review and editing. **Peter Dovč:** conceptualization, methodology, supervision, funding acquisition, project administration, writing – review and editing. **Matjaž Mesarič:** resources, writing – review and editing. **Marko Cotman:** writing – review and editing, resources.

## Funding

This work was supported by Javna Agencija za Raziskovalno Dejavnost RS, J4‐50133, P4‐0220.

## Conflicts of Interest

The authors declare no conflicts of interest.

## Supporting information


**Figure S1:** Pedigree completeness and number of offspring for sampled current breeding animals in Lipica.

**Figure S2:** Heatmap of Nei genetic distances; darker color means a bigger Nei distance.

**Figure S3:** Proportion of the autosomal genome in ROH (FROH) for each length category.

**Figure S4:** Comparison of FPED and FSTR for genotyped horses; the color of the bullet represents the longest ancestral path (LAP).

**Figure S5:** Correlation between Kalinowski's new inbreeding coefficient and ROH‐based inbreeding coefficient calculated for islands longer than 16 Mbp.

**Table S1:** The estimated portion of genetic variability explained by 13 important ancestors that explain about 50% of the gene pool in the pedigree (M—male, F—female).

**Table S2:** Summary statistics of ROH based on five length classes.

**Table S3:** Genomic regions corresponding to ROH islands identified in the Lipica Stud population and annotated using Ensembl BioMart.

**Table S4:** Candidate genes located within ROH islands that have been previously associated with biological functions or traits in other mammalian species.

## Data Availability

The SNP and microsatellite genotype data generated and analysed in this study are publicly available in the Dryad Digital Repository: “SNP and microsatellite genotype data for the characterization of the Lipizzan horse population from Lipica Stud” (https://doi.org/10.5061/dryad.s4mw6m9nb).
