# Supplementary material for: Characterization of Lipizzan Horse Population From Lipica Stud Using Molecular and Pedigree Data
Source: Anim Genet. 2026 Aug 12;57(4):e70182. doi: 10.1002/age.70182 (PMC13467334; doi:10.1002/age.70182)
Supplement: Supplementary file 1 [file AGE-57-0-s004.docx]

Table S2. Summary statistics of ROH based on five length classes.

|  | **Number of ROH** | | **Length of SUM of ROH in Mb** | | **Length of ROH in Mb** | |
| --- | --- | --- | --- | --- | --- | --- |
| **Category** | **Mean number** | **SD** | **Mean length*** | **SD** | **Mean length** | **SD** |
| 1 – 2 Mb | 46.099 | 6.688 | 63.264 | 9.604 | 1.372 | 0.286 |
| 2 – 4 Mb | 24.120 | 4.954 | 68.114 | 14.588 | 2.824 | 0.578 |
| 4 – 8 Mb | 16.648 | 4.231 | 93.585 | 24.186 | 5.621 | 1.125 |
| 8 – 16 Mb | 7.910 | 3.046 | 85.320 | 34.028 | 10.787 | 2.174 |
| > 16 Mb | 2.754 | 1.620 | 63.899 | 41.119 | 23.205 | 7.771 |
| ALL | 97.531 |  | 374.182 |  | 3,837 |  |

*Mean length of SUM of all ROH by sample in the category
