# Supplementary material for: Characterization of Lipizzan Horse Population From Lipica Stud Using Molecular and Pedigree Data
Source: Anim Genet. 2026 Aug 12;57(4):e70182. doi: 10.1002/age.70182 (PMC13467334; doi:10.1002/age.70182)
Supplement: Supplementary file 2 [file AGE-57-0-s005.docx]

Table S4. Candidate genes located within ROH islands that have been previously associated with biological functions or traits in other mammalian species.

| **Chromosome** | **Gene** | **Description** | **Species** | **Reference** |
| --- | --- | --- | --- | --- |
| 1 | *BMPR1A* | Plays a role in cell differentiation. | human | (Ihle et al. 2020) |
| 1 | *MMRN2* | A unique endothelial-specific extracellular matrix protein that has been implicated in angiogenesis and tumour progression. | human | (Khan et al. 2017) |
| 1 | *OPN4* | Sleep Modulator with Direct Effects of Light. | mice | (Tsai et al. 2009) |
| 1 | *SHLD2* | Co‐operates with *REV7* to coordinate DNA double‐strand break repair pathway choice. | human | (Findlay et al. 2018) |
| 1 | *SNCG* | Biomarker for the diagnosis of bladder cancer. | human | (Liu et al. 2016) |
| 2 | *CAMTA1* | Required for long-term memory formation in mice. | mice | (Bas-Orth et al. 2016) |
| 2 | *CXXC4* | Associate with tamoxifen resistance. | human, mice | (Fu et al. 2020) |
| 3 | *ACSF3* | A risk gene in patients with rheumatoid arthritis. | human | (Julià et al. 2017) |
| 5 | *BRDT* | Belongs to the BET protein family, which includes transcriptional regulators of gene expression through recognition of the lysine acetylation of histone H4. | human, mice | (Wu et al. 2022) |
| 7 | *GUCY1A2* | Associated with rheumatoid arthritis. | human | (Gang et al. 2017) |
| 8 | *KSR2* | An obesity gene with effect on trabecular bone volume and bone marrow adiposity. | mouse | (Gomez et al. 2022) |
| 11 | *ABI3* | Related to neurocognitive development. | human | (Dementieva et al. 2023) |
| 13 | *CHTF18* | A component of the conserved Replication Factor C-like complex, in male fertility and gametogenesis and plays crucial roles in female meiosis and oocyte development. | mice | (Holton et al. 2020) |
| 14 | *SPRY4* | Involved in melanoma metastasis and survival rate of melanoma patients. | human | (Grilz-Seger et al. 2019) |
| 16 | *DHX30* | Associated with neurodevelopmental disorders. | human | (Mannucci et al. 2021) |
| 16 | *KIF9* | Important for progressive motility in mouse spermatozoa. | mice | (Miyata et al. 2020) |
| 16 | *SMARCC1* | Plays an essential role in early mouse development. | human, mice | (Uzun et al. 2009) |
| 17 | *BRCA2* | Carcinoma | fishing cat, dog, human | (Flisikowski et al. 2017; Carroll et al. 2024) |
| 25 | *CD72* | Immune system associated. | mice | (Cole 2005) |
| 30 | *PARP1* | Effective in minimizing cell death and inflammation. | human, mice | (Douglas et al. 2015) |

**Supplementary references for Table S4:**

1.	Bas-Orth C., Tan Y.-W., Oliveira A.M.M., Bengtson C.P. & Bading H. (2016) The calmodulin-binding transcription activator CAMTA1 is required for long-term memory formation in mice. Learning & Memory 23, 313-21.

2.	Carroll R.A., Rice E.S., Murphy W.J., Lyons L.A., Thibaud-Nissen F., Coghill L.M., Swanson W.F., Terio K.A., Boyd T. & Warren W.C. (2024) A chromosome-scale fishing cat reference genome for the evaluation of potential germline risk variants. Scientific Reports 14.

3.	Cole C.J. (2005) *Genetic and epigenetic factors in mouse radiation induced leukaemogenesis*. University of Leicester (United Kingdom).

4.	Dementieva N., Nikitkina E., Shcherbakov Y., Nikolaeva O., Mitrofanova O., Ryabova A., Atroshchenko M., Makhmutova O. & Zaitsev A. (2023) The Genetic Diversity of Stallions of Different Breeds in Russia. Genes 14, 1511.

5.	Douglas H.F., Southwood L.L., Meyer‐Ficca M.L., Hart S.K. & Meyer R.G. (2015) The activity and inhibition of poly(ADP‐ribose) polymerase‐1 in equine peripheral blood mononuclear cells in vitro. Journal of Veterinary Emergency and Critical Care 25, 528-37.

6.	Findlay S., Heath J., Luo V.M., Malina A., Morin T., Coulombe Y., Djerir B., Li Z., Samiei A., Simo‐Cheyou E., Karam M., Bagci H., Rahat D., Grapton D., Lavoie E.G., Dove C., Khaled H., Kuasne H., Mann K.K., Klein K.O., Greenwood C.M., Tabach Y., Park M., Côté J.F., Masson J.Y., Maréchal A. & Orthwein A. (2018) *SHLD2*/*FAM35A* co‐operates with *REV7* to coordinate DNA double‐strand break repair pathway choice. The EMBO Journal 37, e100158.

7.	Flisikowski K., Flisikowska T., Sikorska A., Perkowska A., Kind A., Schnieke A. & Switonski M. (2017) Germline gene polymorphisms predisposing domestic mammals to carcinogenesis. Veterinary and Comparative Oncology 15, 289-98.

8.	Fu Y., Wang Z., Luo C., Wang Y., Zhong X. & Zheng H. (2020) Downregulation of CXXC Finger Protein 4 Leads to a Tamoxifen-resistant Phenotype in Breast Cancer Cells Through Activation of the Wnt/β-catenin Pathway. Transl Oncol 13, 423-40.

9.	Gang X., Sun Y., Li F., Yu T., Jiang Z., Zhu X., Jiang Q. & Wang Y. (2017) Identification of key genes associated with rheumatoid arthritis with bioinformatics approach. Medicine (Baltimore) 96, e7673.

10.	Gomez G.A., Rundle C.H., Xing W., Kesavan C., Pourteymoor S., Lewis R.E., Powell D.R. & Mohan S. (2022) Contrasting effects of Ksr2, an obesity gene, on trabecular bone volume and bone marrow adiposity. eLife 11, e82810.

11.	Grilz-Seger G., Druml T., Neuditschko M., Dobretsberger M., Horna M. & Brem G. (2019) High-resolution population structure and runs of homozygosity reveal the genetic architecture of complex traits in the Lipizzan horse. BMC Genomics 20.

12.	Holton R.A., Harris A.M., Mukerji B., Singh T., Dia F. & Berkowitz K.M. (2020) CHTF18 ensures the quantity and quality of the ovarian reserve†. Biology of Reproduction 103, 24-35.

13.	Ihle C.L., Straign D.M., Provera M.D., Novitskiy S.V. & Owens P. (2020) Loss of Myeloid BMPR1a Alters Differentiation and Reduces Mouse Prostate Cancer Growth. Frontiers in Oncology 10.

14.	Julià A., Absher D., López-Lasanta M., Palau N., Pluma A., Waite Jones L., Glossop J.R., Farrell W.E., Myers R.M. & Marsal S. (2017) Epigenome-wide association study of rheumatoid arthritis identifies differentially methylated loci in B cells. Human molecular genetics 26, 2803-11.

15.	Khan K.A., Naylor A.J., Khan A., Noy P.J., Mambretti M., Lodhia P., Athwal J., Korzystka A., Buckley C.D., Willcox B.E., Mohammed F. & Bicknell R. (2017) Multimerin-2 is a ligand for group 14 family C-type lectins CLEC14A, CD93 and CD248 spanning the endothelial pericyte interface. Oncogene 36, 6097-108.

16.	Liu C., Shi B., Hao C., Wang Q., Lv Q., Xing N., Shou J., Qu L., Gao Y., Qin C., Zhao J. & Shou C. (2016) Urine gamma-synuclein as a biomarker for the diagnosis of bladder cancer. Oncotarget 7, 43432-41.

17.	Mannucci I., Dang N.D.P., Huber H., Murry J.B., Abramson J., Althoff T., Banka S., Baynam G., Bearden D., Beleza-Meireles A., Benke P.J., Berland S., Bierhals T., Bilan F., Bindoff L.A., Braathen G.J., Busk Ø.L., Chenbhanich J., Denecke J., Escobar L.F., Estes C., Fleischer J., Groepper D., Haaxma C.A., Hempel M., Holler-Managan Y., Houge G., Jackson A., Kellogg L., Keren B., Kiraly-Borri C., Kraus C., Kubisch C., Le Guyader G., Ljungblad U.W., Brenman L.M., Martinez-Agosto J.A., Might M., Miller D.T., Minks K.Q., Moghaddam B., Nava C., Nelson S.F., Parant J.M., Prescott T., Rajabi F., Randrianaivo H., Reiter S.F., Schuurs-Hoeijmakers J., Shieh P.B., Slavotinek A., Smithson S., Stegmann A.P.A., Tomczak K., Tveten K., Wang J., Whitlock J.H., Zweier C., Mcwalter K., Juusola J., Quintero-Rivera F., Fischer U., Yeo N.C., Kreienkamp H.-J. & Lessel D. (2021) Genotype–phenotype correlations and novel molecular insights into the DHX30-associated neurodevelopmental disorders. Genome Medicine 13.

18.	Miyata H., Shimada K., Morohoshi A., Oura S., Matsumura T., Xu Z., Oyama Y. & Ikawa M. (2020) Testis‐enriched kinesin KIF9 is important for progressive motility in mouse spermatozoa. The FASEB Journal 34, 5389-400.

19.	Tsai J.W., Hannibal J., Hagiwara G., Colas D., Ruppert E., Ruby N.F., Heller H.C., Franken P. & Bourgin P. (2009) Melanopsin as a Sleep Modulator: Circadian Gating of the Direct Effects of Light on Sleep and Altered Sleep Homeostasis in Opn4−/− Mice. PLoS Biology 7, e1000125.

20.	Uzun A., Rodriguez-Osorio N., Kaya A., Wang H., Parrish J.J., Ilyin V.A. & Memili E. (2009) Functional genomics of HMGN3a and SMARCAL1 in early mammalian embryogenesis. BMC Genomics 10, 183.

21.	Wu S., Li X., Shang L., Wu L., Li T., Li P., Ji Z., Hou J., Yin M. & Xu W. (2022) The novel BRDT inhibitor NHWD870 shows potential as a male contraceptive in mice. Acta Biochimica et Biophysica Sinica 54, 1789-800.
