# Supplementary material for: Characterization of Lipizzan Horse Population From Lipica Stud Using Molecular and Pedigree Data
Source: Anim Genet. 2026 Aug 12;57(4):e70182. doi: 10.1002/age.70182 (PMC13467334; doi:10.1002/age.70182)
Supplement: Supplementary file 3 [file AGE-57-0-s008.docx]

Table S3. Genomic regions corresponding to ROH islands identified in the Lipica Stud population and annotated using Ensembl BioMart

| **Chromosome** | **Start position (bp)** | **End position (bp)** | **Animals in ROH** | **Protein-coding genes** |
| --- | --- | --- | --- | --- |
| 1 | 84,265,237 | 84,975,104 | 105 | *SHLD2, SNCG, MMRN2, BMPR1A, LDB3, OPN4, WAPL, GRID1, MIR346* |
| 2 | 43,106,691 | 43,596,174 | 104-121 | *TNFRSF9, UTS2, PER3, VAMP3, CAMTA1* |
|  | 120,537,950 | 121,334,937 | 130 | *CXXC4, TACR3* |
| 3 | 35,893,065 | 37,590,699 | 109-119 | *MVD, SNAI3, CTU2, RNF166, PIEZO1, CDT1, APRT, GALNS, PABPN1L, CBFA2T3, ACSF3, CDH15, ANKRD11, eca-mir-9074, SPG7, RPL13, CPNE7, DPEP1, CHMP1A, CDK10, SPATA2L, VPS9D1, ZNF276, FANCA, SPIRE2, TCF25, MC1R, TUBB3, DEF8, GAS8, DBNDD1, URAH, SLC9B2* |
| 5 | 45,068,182 | 46,367,861 | 135-138 | *CHD1L, FMO5, PRKAB2, PDE4DIP, SEC22B, NOTCH2, ADAM30* |
|  | 69,645,386 | 69,693,724 | 107-114 | *BRDT* |
| 6 | 17,077,595 | 17,818,788 | 107-111 | *DNER, TRIP12, FBXO36, SLC16A14, SP110* |
| 7 | 16,621,859 | 16,785,617 | 109-112 | *GUCY1A2* |
|  | 24,901,636 | 25,101,286 | 108-114 | *-* |
|  | 45,588,946 | 45,785,089 | 109 | *ADGRE3, ZNF333, CLEC17A* |
|  | 46,064,822 | 46,255,923 | 109 | *PRKACA, C7H19orf67, PALM3, MISP3, IL27RA, eca-mir-1271b, RFX1, DCAF15, PODNL1, CC2D1A, BRME1, NANOS3, ZSWIM4, eca-mir-23a, eca-mir-27a, eca-mir-24-1, eca-mir-8992* |
| 8 | 17,430,225 | 19,888,454 | 104-105 | *SUDS3, TAOK3, PEBP1, VSIG10, WSB2, RFC5, KSR2, NOS1, FBXO21, TESC, FBXW8, RNFT2, U6, MED13L* |
|  | 24,575,702 | 25,422,613 | 105-109 | *TMEM120B, RHOF, SETD1B, HPD, PSMD9, CFAP251, BCL7A, MLXIP, IL31, LRRC43, B3GNT4, DIABLO, VPS33A, CLIP1, ZCCHC8, RSRC2, KNTC1, HCAR1, DENR, CCDC62, HIP1R* |
| 9 | 36,935,799 | 37,989,426 | 113 | *SPIDR* |
| 11 | 25,233,252 | 26,166,294 | 111-128 | *ABI3, PHOSPHO1, ZNF652, PHB1, NXPH3, SPOP, FAM117A, KAT7, TAC4, DLX4, DLX3, ITGA3, PDK2, SAMD14, PPP1R9B, SGCA, COL1A1, TMEM92, XYLT2, EME1, MRPL27* |
|  | 28,841,728 | 29,080,732 | 106-144 | *KIF2B, TOM1L1, COX11, STXBP4, HLF, MMD, SMIM36, TMEM100, PCTP, ANKFN1, NOG, C11H17orf67, DGKE, COIL, SCPEP1, AKAP1, MSI2, CCDC182, MRPS23, CUEDC1, VEZF1, SRSF1, OR4D1, OR4D2H, EPX, MKS1, LPO, MPO, TSPOAP1, MIR142, SUPT4H1, RNF43, HSF5, MTMR4, TEX14, SNORD3D, U3, U1, RAD51C, PPM1E, TRIM37, U6, eca-mir-454, eca-mir-301a, PRR11, SMG8, GDPD1* |
| 13 | 43,059,697 | 43,775,185 | 108-112 | *GNG13, CHTF18, RPUSD1, MSLNL, CIAO3, HAGHL, CCDC78, METRN, FBXL16, WDR24, JMJD8, STUB1, RHBDL1, RHOT2, WDR90, METTL26, WFIKKN1, RAB40C, PIGQ, PRR35, CAPN15, eca-mir-9140, RAB11FIP3, DECR2, PGAP6, NME4, MRPL28, AXIN1, PDIA2, ARHGDIG, RGS11, FAM234A, LUC7L, HBQ1, HBZ, NPRL3, MPG, RHBDF1, POLR3K, IL9R* |
| 14 | 33,331,189 | 34,678,352 | 106-157 | *ARHGAP26, eca-mir-9162, FGF1, SPRY4, NDFIP1, GNPDA1, RNF14, PCDH12, DELE1, PCDH1, ARAP3, eca-mir-9157, FCHSD1* |
| 15 | 2,566,341 | 2,777,342 | 107 | *-* |
| 16 | 40,637,672 | 41,748,489 | 113-120 | *MAP4, DHX30, SMARCC1, ELP6, SCAP, U4, PTPN23, KIF9, KLHL18, SETD2, eca-mir-9174, NRADD, NBEAL2, CCDC12, PTH1R, MYL3, PRSS45, TMIE, ALS2CL, FAM240A* |
|  | 45,576,210 | 47,213,753 | 104-113 | *TRAK1, ULK4, CTNNB1, ZNF621, ZNF620, ZNF619, RPL14, ENTPD3, EIF1B, MYRIP* |
| 17 | 10,864,297 | 12,087,588 | 105-134 | *RXFP2, FRY, ZAR1L, N4BP2L1, BRCA2, N4BP2L2, PDS5B, U6* |
| 18 | 565,330 | 1,488,976 | 120-123 | *FAM168B, PLEKHB2* |
| 25 | 229,064 | 1,134,434 | 108-117 | *PHF24, U6, DNAJB5, C25H9orf131, VCP, FANCG, PIGO, STOML2, ATOSB, UNC13B, RUSC2, CIMIP2B, CD72, TESK1, CCDC107, RNase_MRP, ARHGEF39, CA9, TPM2, TLN1, CREB3, GBA2, RGP1, NPR2, SPAG8, HINT2, FAM221B, TMEM8B* |
|  | 5,746,742 | 6,747,435 | 125-130 | *COL15A1, TGFBR1, ALG2, SEC61B, NR4A3, STX17, ERP44, INVS* |
|  | 29,075,705 | 29,187,610 | 105 | *NR5A1, NR6A1* |
| 30 | 7,065,824 | 9,611,340 | 107-115 | *PSEN2, ITPKB, STUM, PARP1, LIN9, MIXL1, ACBD3, H3-3A, SDE2, PYCR2, TMEM63A, EPHX1, SRP9, ENAH, LBR, DNAH14, CNIH3, WDR26, CNIH4, NVL, DEGS1, FBXO28, TP53BP2, eca-mir-9126* |
|  | 29,145,408 | 30,136,586 | 104-109 | *DDX59, CAMSAP2, INAVA, CACNA1S, ASCL5, PKP1, TNNT2, LAD1, TNNI1, PHLDA3, CSRP1, NAV1, IPO9* |
