# Supplementary material for: Characterization of Lipizzan Horse Population From Lipica Stud Using Molecular and Pedigree Data
Source: Anim Genet. 2026 Aug 12;57(4):e70182. doi: 10.1002/age.70182 (PMC13467334; doi:10.1002/age.70182)
Supplement: Supplementary file 4 [file AGE-57-0-s009.docx]

Table S1. The estimated portion of genetic variability explained by 13 important ancestors that explain about 50% of the gene pool in the pedigree (M – male, F – female).

| **NAME** | **Year of birth** | **Country** | **Sex** | **Cumulative portion**  **of genetic variability**  **explained by included ancestors (%)** | **The portion**  **of genetic variability**  **explained by the ancestor (%)** |
| --- | --- | --- | --- | --- | --- |
| FAVORY RATISBONA II | 1829 | SI | M | 7.98 | 7.98 |
| FAVORY ONEROSA | 1819 | SI | M | 14.52 | 6.54 |
| NEAPOLITANO AQUILEJA | 1820 | SI | M | 19.68 | 5.16 |
| MAESTOSO ERGA XXXVI | 1838 | SI | M | 24.24 | 4.56 |
| OX GAZLAN | 1840 | SI | M | 28.37 | 4.13 |
| SIGLAVY ALEA | 1846 | SI | M | 32.08 | 3.71 |
| CONVERSANO ERGA | 1848 | SI | M | 35.72 | 3.64 |
| PLUTO ALEA | 1853 | SI | M | 39.33 | 3.61 |
| SLAVINA II | 1847 | SI | F | 42.30 | 2.97 |
| GROCZANA II | 1817 | SI | F | 44.93 | 2.63 |
| LIPP I NORWEGIA | 1797 | SI | M | 47.17 | 2.24 |
| 1 PLUTO FANTASCA-10 | 1895 | SI | M | 48.85 | 1.68 |
| MAESTOSO MASCULA | 1874 | SI | M | 50.42 | 1.57 |
